# Defining Phenotype, Tropism, and Retinal Gene Therapy Using Adeno-Associated Viral Vectors (AAVs) in New-Born Brown Norway Rats with a Spontaneous Mutation in *Crb1*

**DOI:** 10.3390/ijms22073563

**Published:** 2021-03-30

**Authors:** Nanda Boon, C. Henrique Alves, Aat A. Mulder, Charlotte A. Andriessen, Thilo M. Buck, Peter M. J. Quinn, Rogier M. Vos, Abraham J. Koster, Carolina R. Jost, Jan Wijnholds

**Affiliations:** 1 Department of Ophthalmology, Leiden University Medical Center (LUMC), Albinusdreef 2, 2333 ZA Leiden, The Netherlands; n.boon@lumc.nl (N.B.); chalves@fmed.uc.pt (C.H.A.); C.A.Andriessen@lumc.nl (C.A.A.); T.M.Buck@lumc.nl (T.M.B.); pq2138@cumc.columbia.edu (P.M.J.Q.); rogier_vos@hotmail.com (R.M.V.); 2 Department of Cell & Chemical Biology, Leiden University Medical Center (LUMC), 2300 RC Leiden, The Netherlands; A.A.Mulder@lumc.nl (A.A.M.); A.J.Koster@lumc.nl (A.J.K.); C.R.Jost@lumc.nl (C.R.J.); 3 Netherlands Institute for Neuroscience, Institute of the Royal Netherlands Academy of Arts and Sciences (KNAW), Meibergdreef 47, 1105 BA Amsterdam, The Netherlands

**Keywords:** CRB1, CRB2, Crumbs homologue 1, retinitis pigmentosa, AAV tropism, AAV-mediated gene therapy

## Abstract

Mutations in the Crumbs homologue 1 (*CRB1*) gene cause inherited retinal dystrophies, such as early-onset retinitis pigmentosa and Leber congenital amaurosis. A Brown Norway rat strain was reported with a spontaneous insertion-deletion (indel) mutation in exon 6 of *Crb1*. It has been reported that these *Crb1* mutant rats show vascular abnormalities associated with retinal telangiectasia and possess an early-onset retinal degenerative phenotype with outer limiting membrane breaks and focal loss of retinal lamination at 2 months of age. Here, we further characterized the morphological phenotype of new-born and adult *Crb1* mutant rats in comparison with age-matched Brown Norway rats without a mutation in *Crb1*. A significantly decreased retinal function and visual acuity was observed in *Crb1* mutant rats at 1 and 3 months of age, respectively. Moreover, in control rats, the subcellular localization of canonical CRB1 was observed at the subapical region in Müller glial cells while CRB2 was observed at the subapical region in both photoreceptors and Müller glial cells by immuno-electron microscopy. CRB1 localization was lost in the *Crb1* mutant rats, whereas CRB2 was still observed. In addition, we determined the tropism of subretinal or intravitreally administered AAV5-, AAV9- or AAV6-variant ShH10^Y445F^ vectors in new-born control and *Crb1* mutant rat retinas. We showed that subretinal injection of AAV5 and AAV9 at postnatal days 5 (P5) or 8 (P8) predominantly infected the retinal pigment epithelium (RPE) and photoreceptor cells; while intravitreal injection of ShH10^Y445F^ at P5 or P8 resulted in efficient infection of mainly Müller glial cells. Using knowledge of the subcellular localization of CRB1 and the ability of ShH10^Y445F^ to infect Müller glial cells, canonical h*CRB1* and h*CRB2* AAV-mediated gene therapy were explored in new-born *Crb1* mutant rats. Enhanced retinal function after gene therapy delivery in the *Crb1* rat was not observed. No timely rescue of the retinal phenotype was observed using retinal function and visual acuity, suggesting the need for earlier onset of expression of recombinant hCRB proteins in Müller glial cells to rescue the severe retinal phenotype in *Crb1* mutant rats.

## 1. Introduction

Retinitis pigmentosa (RP) and Leber congenital amaurosis (LCA) are inherited retinal degenerative diseases causing progressive vision loss, ultimately leading to blindness. Mutations in the Crumbs homolog 1 (*CRB1*) gene is a frequent cause of these retinal dystrophies in humans [1]. The *CRB1* gene, mapped to chromosome 1q31.3, encodes a large transmembrane protein and belongs to the Crumbs (CRB) family, with family members CRB2 and CRB3. CRB proteins are located in the subapical region above adherens junctions at the outer limiting membrane (OLM), where it can interact with, amongst others, PALS1 to form the canonical Crumbs complex [2,3,4]. *Crb1* knockout (*Crb1*^KO^) mouse models show a mild retinal degeneration with OLM disruptions and ectopic rows of photoreceptor cell nuclei in the photoreceptor segment layers from postnatal day 14 (P14) [5]. Concomitant loss of *Crb2* in *Crb1*^KO^ mice results in a more severe RP or LCA phenotype, depending on which cell type lacks *Crb2* [6,7,8,9]. Currently, there is no treatment available for *CRB1*-related retinal dystrophies.

A Brown Norway rat strain was described with an inherited retinal degenerative phenotype caused by a spontaneous in frame insertion-deletion (indel) in exon 6 of the *Crb1* gene [10]. These *Crb1* mutant rats, expressing an alternative CRB1^INDEL^ protein, exhibit an early-onset loss of retinal function from 3 weeks of age. In addition, the first signs of retinal degeneration were observed from P15, including OLM disruptions and ectopic rows of photoreceptor cell nuclei in the photoreceptor segment layers. At older ages, these disruptions progress and ultimately lead to a focal loss of retinal lamination [10]. Because of its naturally occurring mutation and early-onset severe retinal phenotype, these rats are a potential attractive animal model for the development of gene therapy. Currently, gene therapy using adeno-associated viral vectors (AAVs) is the leading platform of gene delivery for the treatment of retinal dystrophies because of its low toxicity and ability to target both dividing and non-dividing cells [11]. In addition, different AAV capsids display distinct cell tropisms, making it possible to target different cell types. Moreover, previous mouse gene supplementation studies with AAV expressing human *CRB2* (h*CRB2*) have shown to preserve the retinal morphology and function in *Crb1* RP mouse models [12,13]. Altogether, this indicates the potential use of AAV-mediated gene therapy for *CRB1*-related retinal dystrophies.

Here, we perform a thorough characterization of the morphological phenotype of new-born and adult *Crb1* mutant rats and its effect on retinal function and visual acuity in comparison with age-matched control rats. Using immuno-electron-microscopy, we show that, in control rats, canonical CRB1 localizes specifically in Müller glial cells (MGCs) at the subapical region adjacent to the adherens junctions at the outer limiting membrane (OLM), whereas the *Crb1* mutant rats barely express detectable levels of CRB1^INDEL^ at the subapical region of MGCs. In turn, CRB2 is localized at the subapical region in MGCs and photoreceptors of both control and *Crb1* mutant rats. Next, we describe the tropism of three different AAV serotypes (AAV5-, AAV9- and AAV6-variant ShH10Y445F) expressing *GFP* driven by the cytomegalovirus (CMV) promoter in new-born control and *Crb1* mutant rat retinas. Tropism data at P5 and P8 shows that subretinal injection of AAV5.CMV.*GFP* and AAV9.CMV.*GFP* mainly transduces photoreceptors and retinal pigment epithelium (RPE), whereas intravitreal injection at P5 or P8 of ShH10^Y445F^.CMV.*GFP* efficiently transduces MGCs. Based on this knowledge, AAV-mediated h*CRB1* and h*CRB2* gene therapy were explored in P5 *Crb1* mutant rats.

## 2. Results

### 2.1. The Spontaneous Mutation in Crb1 in Brown Norway Rats Leads to Retinal Dysfunction and Vision Impairment

**Figure 1:**
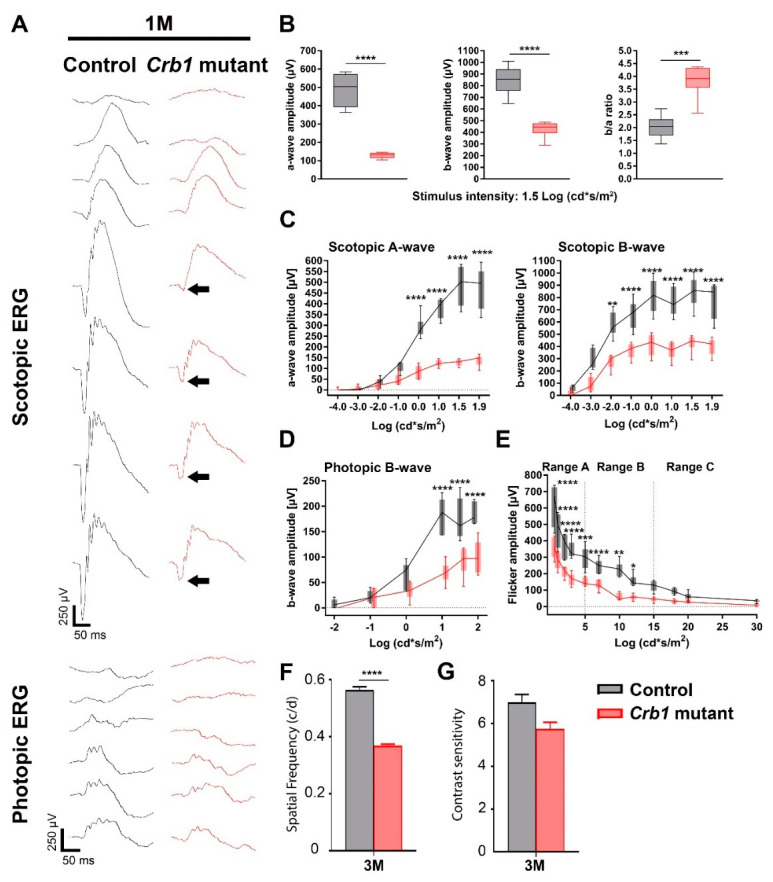
Significantly reduced retinal function in the *Crb1* mutant compared to age-matched control rats. (**A**) Scotopic and photopic single-flash intensity series from representative rats at one month of age. The attenuated scotopic a-wave of the *Crb1* mutant rats is indicated with the black arrow. (**B**) Quantitative analysis of the scotopic a-wave, b-wave and b-wave/a-wave amplitude ratio (b/a ratio). Quantitative analysis of the scotopic a-wave and b-wave (**C**), the photopic b-wave (**D**) and the flicker amplitude response (**E**). Boxes indicate the 25 and 75% quantile range and whiskers indicate the 5 and 95% quantiles, and the intersection of line and error bar indicates the median of the data (box-and-whisker plot). Decreased visual function in 3 months of age *Crb1* mutant rats measured using the optokinetic head tracking response (OKT) spatial frequency (**F**) and OKT contrast sensitivity (**G**). Number of animals used for ERG: *n* = 6 for control and *Crb1* mutant rats; and for OKT: control *n* = 4 and *Crb1* mutant = 10. Mean ± SEM. * *p* < 0.05; ** *p* < 0.01; *** *p* < 0.001; **** *p* < 0.0001.

To study the retinal function of the Brown Norway rats with a spontaneous mutation in the *Crb1* gene, we performed electroretinography (ERG) in 1-, 3- and 5-month-old *Crb1* mutant rats compared to age-matched control Brown Norway rats without a mutation in *Crb1*. One-month-old *Crb1* mutant rats showed significantly reduced a- and b-wave responses under scotopic conditions (Figure 1A,C). In addition, a significant reduction in photopic b-wave was observed (Figure 1C,D). Finally, there was a significantly reduced flicker amplitude response in *Crb1* mutant rats compared to age-matched control rats in range A and B, indicating aberrations in the rod pathway and cone pathway, respectively (Figure 1E). At 3 and 5 months of age, the retinal degeneration continued in the *Crb1* mutant rats and the ERG response was further reduced compared to the age-matched control rats (Figure S1).

In addition, the visual function was determined using an optomotor response test (optokinetic head tracking response (OKT)). The OKT measures spatial frequency threshold, also called visual acuity, by systematically increasing the spatial frequency of the grating at 100% contrast until the animals no longer perform head tracking. In addition, contrast sensitivity can also be measured, where the minimum contrast that generated a tracking response was identified over a range of spatial frequencies [14,15]. Three-month-old *Crb1* mutant rats showed a significantly decreased OKT spatial frequency, indicating a loss in visual function compared to control rats (Figure 1F,G). In summary, *Crb1* mutant rats presents a severely decreased retinal function and visual function within three months of age.

### 2.2. First Signs of Retinal Degeneration in Crb1 Mutant Rats Are Observed from Postnatal Day 10

**Figure 2:**
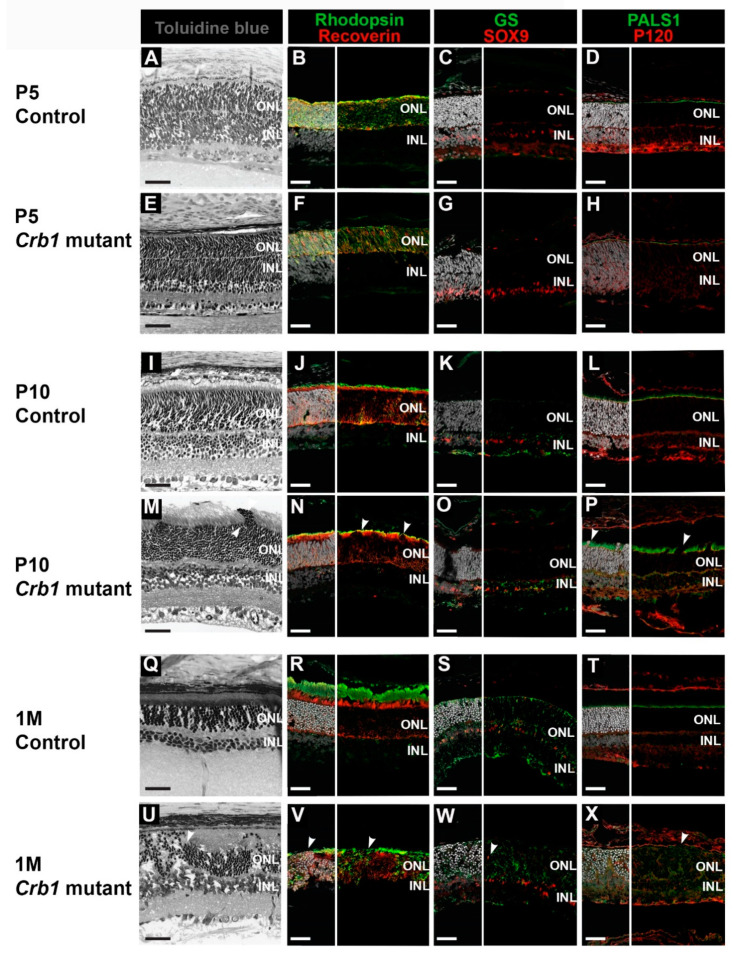
Retinal degeneration is observed from postnatal day 10 retinas of *Crb1* mutant rats. Light microscopy of toluidine blue-stained retinal sections from P5, P10 and 1-month-old control (**A**,**I**,**Q**) and *Crb1* mutant rats (**E**,**M**,**U**). Immunohistochemistry staining of P5, P10 and 1-month-old retinal sections, with recoverin and rhodopsin (**B**,**F**,**J**,**N**,**R**,**V**), glutamine synthetase (GS) and SOX9 (**C**,**G**,**K**,**O**,**S**,**W**) and PALS1 with P120-catenin (**D**,**H**,**L**,**P**,**T**,**X**). No abnormalities were observed in the control retina (**A**–**D**,**I**–**L**,**Q**–**T**). In the *Crb1* mutant rats, all retinal layers were formed and properly laminated at P5 (**E**–**H**). From P10 onwards, degeneration at the foci was observed in the *Crb1* mutant rat retina by photoreceptor nuclei protrusions into the photoreceptor segment layers, misplaced Müller glial cells (MGCs) and disruption of the outer limiting membrane (OLM) (**M**–**P**,**U**–**X**; arrowheads). At least *n* = 2 eyes were used per time point. GCL, ganglion cell layer; INL, inner nuclear layer; ONL, outer nuclear layer; RPE, retinal pigment epithelium. Scale bar: 40 µm.

To study the morphological phenotype of new-born *Crb1* mutant rats, histological analysis of retina sections was performed and compared to age-matched controls. No abnormalities in the retinal development and lamination were observed in P5 *Crb1* mutant rats compared with the control (Figure 2A,E). In addition, immunohistochemical analysis of P5 *Crb1* mutant and control rat retinas showed similar localization of photoreceptors in the outer nuclear layer (ONL), indicated by recoverin and rhodopsin staining (Figure 2B,F); MGC localization within the inner nuclear layer (INL), indicated by SOX9 staining (Figure 2C,G); and a continuous OLM, indicated by localization of subapical region marker PALS1 and adherens junctions marker P120-catenin (Figure 2D,H). In accordance with previous data [16], glutamine synthetase (GS), a mature MGC marker, was not yet fully expressed in the P5 control nor *Crb1* mutant retinas (Figure 2C,G).

In contrast to previous findings, where the phenotype was first observed at P15 [10], here we observed focal disruptions already at P10 in the *Crb1* mutant rat retina (Figure 2M). The phenotype includes OLM breaks (Figure 2P, arrowhead) and photoreceptor cell nuclei protrusions into the photoreceptor segment layers (Figure 2N). Interestingly, PALS1 staining is more diffuse in P10 *Crb1* mutant rats in comparison to age-matched controls (Figure 2L,P). In addition, at P10, low levels of GS were detectable and appeared similar between control and *Crb1* mutant retinas (Figure 2K,O). One-month-old control rat retinas present mature photoreceptor cells, MGCs with radial processes throughout the retinal layers and a continuous OLM (Figure 2Q–T). In contrast, the retina of *Crb1* mutant rats is severely disorganized (Figure 2U), characterized by, at the foci, intermingling of nuclei from the INL and ONL and loss of photoreceptor inner and outer segments (Figure 2V, arrowhead), misplaced SOX9-positive MGC nuclei and disorganized radial MGC processes (Figure 2W). In addition, we observed OLM breaks indicated by disruptions in PALS1 and P120-catenin staining (Figure 2X, arrowhead). Similar results were obtained in 2-months-old *Crb1* mutant rats, where focal loss of retinal lamination, OLM disruptions and photoreceptor alterations were observed [10]. Altogether, these results highlight the early-onset degenerative retinal phenotype in *Crb1* mutant compared to control rats.

### 2.3. The Crb1 Mutant Rat Retina Develops a Progressive Lack of Retinal Lamination

**Figure 3:**
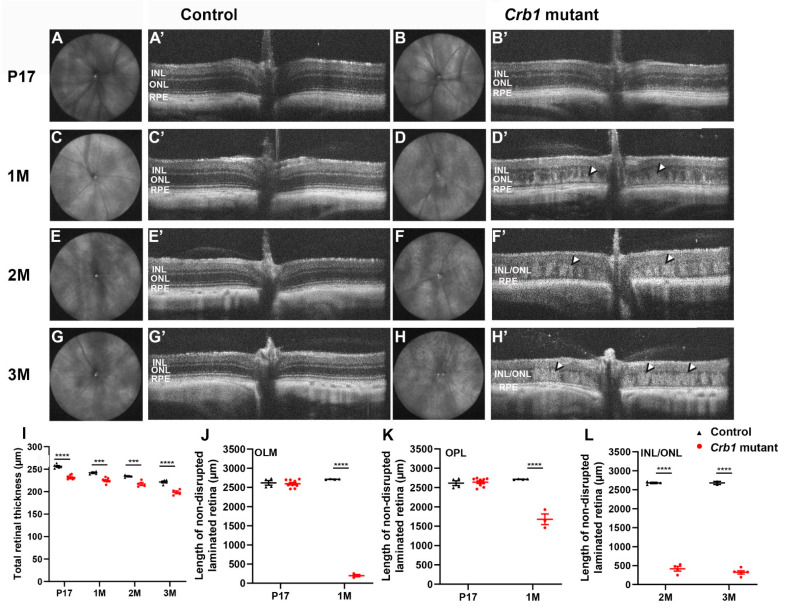
In vivo imaging of *Crb1* mutant rats show increased retinal degeneration over time. Volume intensity projection (VIP) of the P17 and 1, 2 and 3 months of age control (**A**,**C**,**E**,**G**) and *Crb1* mutant (**B**,**D**,**F**,**H**) rat retinas; and SD-OCT B-scans of the P17 and 1, 2 and 3 months of age control (**A’**,**C’**,**E’**,**G’**) and *Crb1* mutant rat retinas (**B’**,**D’**,**F’**,**H’**). Quantifications of total retinal thickness (**I**), length of non-disrupted laminated retina at the OLM in P17 and 1-month-old control and *Crb1* mutant rats (**J**), at the OPL (**K**), and in 2 and 3 months of age rats at the INL/ONL (**L**). Arrowheads indicate regions of retinal disorganization. Number of animals used for P17 (control *n* = 6, *Crb1* mutant = 9), 1M (control *n* = 4, *Crb1* mutant *n* = 9), 2M (control *n* = 4, *Crb1* mutant *n* = 4) and 3M (control *n* = 2, *Crb1* mutant *n* = 5). Values are presented as the mean ± SEM. *** *p* < 0.001, **** *p* < 0.0001. INL = inner nuclear layer; ONL = outer nuclear layer; OPL = outer plexiform layer; RPE = retinal pigment epithelium.

Using SD-OCT imaging, the retinal degeneration was followed in vivo at P17 and 1, 2 and 3 months of age *Crb1* mutant rats in comparison with control rats. All retinal layers were correctly laminated at all ages measured in the control rat (Figure 3A’,C’,E’,G’). At P17, retinal lamination in *Crb1* mutant rats appeared similar to control rats (Figure 3B’). At one-month-old *Crb1* mutant rats, the retinal lamination is affected mainly at the OLM indicated by a hyperreflective ONL (Figure 3D’, arrowhead). Two and three months of age *Crb1* mutant rats show a further increased retinal degeneration, indicated by a hyperreflective INL and ONL, resulting in an unclear distinction between the INL and the ONL (Figure 3F’,H’, arrowhead). Volume intensity projection (VIP) shows disruptions throughout the *Crb1* mutant retina (Figure 3B,D,F,H). Morphological sections of the *Crb1* mutant rats show a similar degeneration, with disruptions at the OLM and intermingling of nuclei of the INL and ONL at 1 and 3 months of age rats (Figure 2M,Q,U).

Quantification of the total retinal thickness at all ages revealed a significant decrease in the *Crb1* mutant compared to control rat retinas (Figure 3I). In addition, the length of disrupted and normal laminated retina was determined in both the control and *Crb1* mutant rat retina at all ages analyzed. At P17, both control and *Crb1* mutant rat retinas mainly showed a normal retinal lamination with a limited number of disruptions at the OLM and the outer plexiform layer (OPL) (Figure 3J,K). At one month of age, a significant increase in the length of degenerated retina were observed, indicated by OLM breaks (Figure 3J) or disruptions at the OPL in the *Crb1* mutant rats (Figure 3K). Finally, at two and three months of age, the length of areas of INL and ONL disruptions increased in the *Crb1* mutant rats (Figure 3L). Altogether, these data demonstrate the early-onset severe degenerative phenotype observed in *Crb1* mutant rats over time.

### 2.4. Ultra-Structural Localization of CRB1 and CRB2 Proteins in the Control and Crb1 Mutant Rat Retina

**Figure 4:**
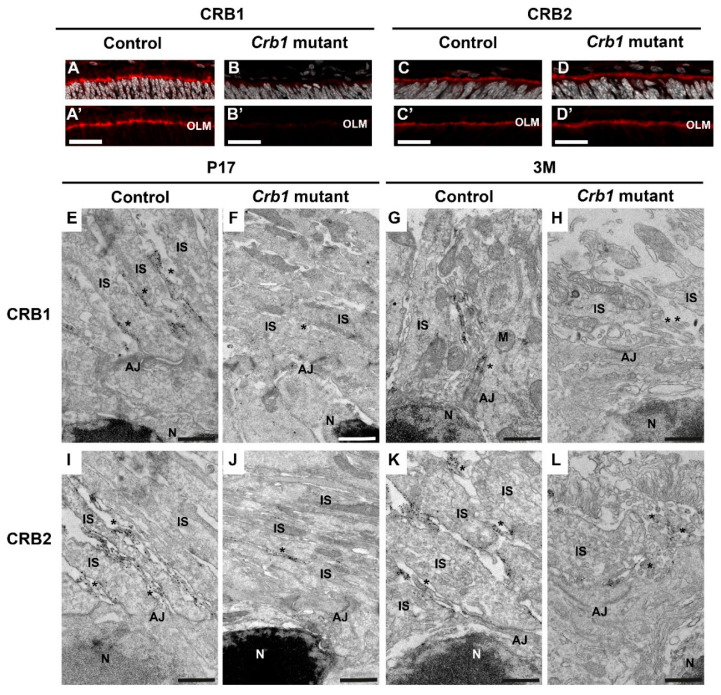
CRB1 is present at the subapical region (SAR) of MGCs in control rats while CRB2 is present at the SAR of both photoreceptors and MGC in control and *Crb1* mutant rats. (**A**–**D**) Immunohistochemical analysis of CRB1 (**A**,**B**) and CRB2 (**C**,**D**) in P5 control and *Crb1* mutant rats at the OLM. (**E**–**L**) Subcellular localization of CRB1 and CRB2 in control and *Crb1* mutant rats using immuno-electron microscopy. CRB1 is present at the SAR of MGC in both P17 (**E**) and 3M control retina (**G**), while the mutant CRB1^INDEL^ variant protein in *Crb1* mutant rats is sporadically detectable in P17 (**F**) and 3M (**H**). F and H show representative images lacking detectable label. CRB2 is present at the SAR of MGCs as well as photoreceptors in both control and *Crb1* mutant rat retinas at both ages (**I**–**L**). *n* = 2 animals used per time point. AJ = adherens junctions; IS = inner segments; INL = inner nuclear layer; M = mitochondria; N = nucleus; OLM = outer limiting membrane; ONL = outer nuclear layer; * = Müller glial villi. Scale bars (**A**–**D**) = 20 µm; (**E**–**L**) = 1 µm.

**Figure 5:**
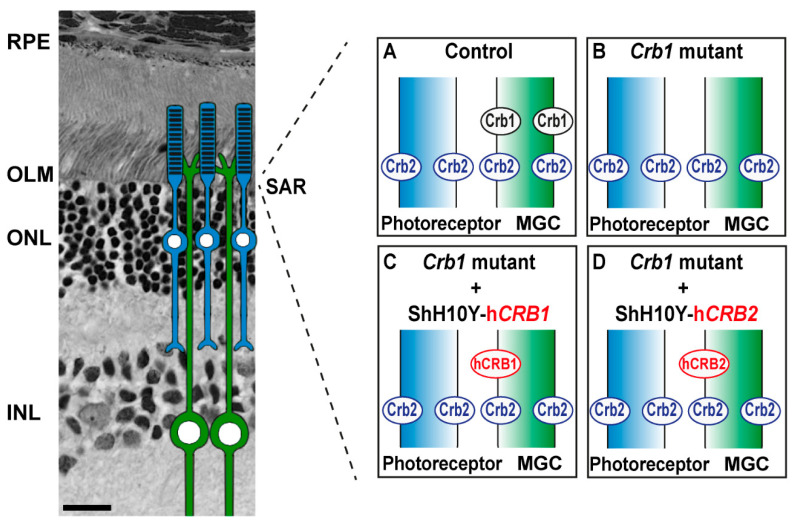
Graphical representation of subcellular localization of Crb1 and Crb2 proteins in Brown Norway rat retina and gene therapy approach using ShH10Y-h*CRB1* and ShH10Y-h*CRB2*. Graphical representation of the subcellular localization of Cb1 and Crb2 in control (**A**) and *Crb1* mutant (**B**) rat retinas. Graphical representation using AAV-mediated gene therapy vectors targeting MGCs using ShH10Y-h*CRB1* (**C**) and ShH10Y-h*CRB2* (**D**). Scale bar: 20 µm. RPE = retinal pigment epithelium; OLM = outer limiting membrane; ONL = outer nuclear layer; INL = inner nuclear layer; SAR = subapical region; MGC = Müller glial cell.

CRB1 and CRB2 localization was studied by immunohistochemistry in the P5 control and *Crb1* mutant rat retinas. Canonical CRB1 was found at the OLM in control rats, while the CRB1^INDEL^ protein was below the detection level in the *Crb1* mutant rat retina (Figure 4A,B). CRB2 was detected at the OLM in both control and *Crb1* mutant rats (Figure 4C,D). Interestingly, the CRB2 staining is more diffuse in the *Crb1* mutant in comparison to control rats (Figure 4C,D). In addition, the onset of retinal function loss in the *Crb1* mutant rats is remarkably faster than in mice lacking CRB1 (*Crb1*^KO^), mice carrying a missense CRB1 mutation (Crb1^KO/C249W^) or the naturally occurring *Crb1* rd8 mouse [3,5,17,18]. Previously, we found that CRB1 or CRB2 proteins localized differently in adult human retina, fetal human retina, human iPSC-derived retinal organoids and non-human-primate retina compared to mouse retina [8,19]. For that reason, we performed immuno-electron microscopy to determine the localization of canonical CRB1 and CRB2 in the control compared to *Crb1* mutant rat retinas. In control rats, CRB1 was abundantly present at the subapical region in MGCs but not photoreceptors at both P17 and 3 months of age (Figure 4E,G and Figure 5A), while the CRB1^INDEL^ variant protein in *Crb1* mutant rats was only sporadically and at very low levels detected at the subapical region in MGCs (Figure 4F,H). For CRB2, a different pattern was observed: CRB2 is present at the subapical region of the MGCs and photoreceptors in both control and *Crb1* mutant retinas (Figure 4I–L and Figure 5B). The cellular localizations of CRB1 and CRB2 are therefore similar in the mouse and rat.

### 2.5. Adeno-Associated Viral Vector (AAV) Tropism in Young Brown Norway Rat Retina

To define the tropism of different AAV capsids, 1 µL of 1 × 10^13^ gc/mL AAV2-CMV-*GFP* expression vectors packaged into three different AAV serotypes (AAV2/5.CMV.*GFP*, AAV2/9.CMV.*GFP* and AAV2/ShH10^Y445F^.CMV.*GFP*, called from now on AAV5, AAV9 and ShH10Y, respectively) were intravitreally or subretinally injected before retinal degeneration in P5 and P8 in control and *Crb1* mutant rat retinas. One month after injection, eyes were collected and analyzed using immunohistochemistry. Differences in retinal tropism were observed using the distinct application methods in new-born control and *Crb1* mutant rats; a summary of the tropism in the different serotypes, routes and time points of delivery is shown in Table 1. Interestingly, no difference in tropism was observed between control and *Crb1* mutant rats, nor the time point of the injection (P5 or P8; Table 1).

**Figure 6:**
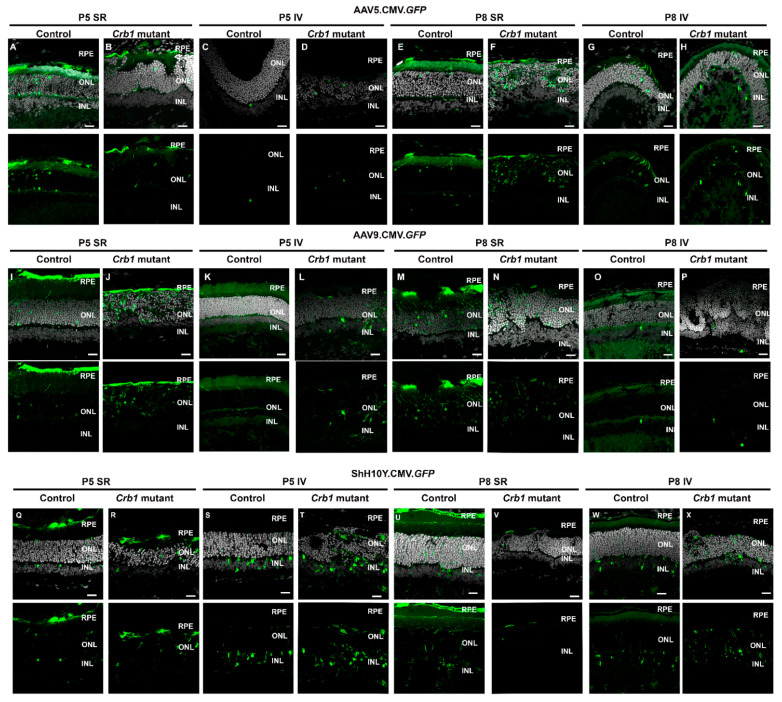
Retinal tropisms of AAV5, AAV9 and ShH0Y in new-born *Crb1* mutant and control rat retinas. P5 or P8 control and *Crb1* mutant rats were injected subretinally (SR) or intravitreally (IV) with either AAV5-, AAV9- or ShH10Y.CMV.*GFP* and analyzed at 1 month of age. Tropism studies show that SR injection of AAV5 and AAV9 mainly infect RPE and photoreceptors at P5 (**A**,**B**,**I**,**J**); similar results were obtained when injected at P8 (**E**,**F**,**M**,**N**). IV injection at P5 of AAV5 and AAV9 show mainly infection of sporadic photoreceptors and some INL cells (**C**,**D**,**K**,**L**); similar results were obtained when injected at P8 (**G**,**H**,**O**,**P**). SR injection of ShH10Y at both P5 and P8 in control and *Crb1* mutant retinas results in infection of RPE, photoreceptors and INL cells (**Q**,**R**,**U**,**V**); analysis of IV injection of ShH10Y at both P5 and P8 show transduction of INL cells (**S**,**T**,**W**,**X**). For each serotype and route of delivery, a dose of ~1 × 10^13^ gc/mL was injected in a volume of 1 µL. Scale bar: 20 µm. Number of animals analyzed: AAV5 control P5 SR *n* = 6, P5 IV *n* = 2, P8 SR *n* = 2, P8 IV *n* = 2, AAV5 *Crb1* mutant P5 SR *n* = 4, P5 IV *n* = 3, P8 SR *n* = 2, P8 IV *n* = 5; AAV9 control P5 SR *n* = 3, P5 IV *n* = 2, P8 SR *n* = 3, P8 IV *n* = 3, AAV9 *Crb1* mutant P5 SR *n* = 4, P5 IV *n* = 3, P8 SR *n* = 2, P8 IV *n* = 3; ShH10Y control P5 SR *n* = 2, P5 IV *n* = 6, P8 SR *n* = 2, P8 IV *n* = 5, ShH10Y *Crb1* mutant P5 SR *n* = 3, P5 IV *n* = 4, P8 SR *n* = 2, P8 IV *n* = 3.

Subretinal injection of AAV5 and AAV9 at both P5 and P8 resulted in predominantly transduced RPE and photoreceptors, whereas intravitreal injection of both serotypes showed transduction of photoreceptors and, to a lesser extent, transduction of INL cells (Figure 6A–P). Furthermore, subretinal injection of ShH10Y demonstrated infection of RPE cells, photoreceptors and MGCs (Figure 6Q,R,U,V), while intravitreal injection of ShH10Y resulted mainly in transduction of MGCs and other cell types in the INL (Figure 6S,T,W,X). Co-staining with GS confirmed the transduction of mainly MGCs with all ShH10Y injections performed (Figure S2).

### 2.6. Intravitreal Delivery of ShH10Y-hCRB1 or ShH10Y-hCRB2 at P5 Does Not Increase the Retinal Function in Crb1 Mutant Rats

**Figure 7:**
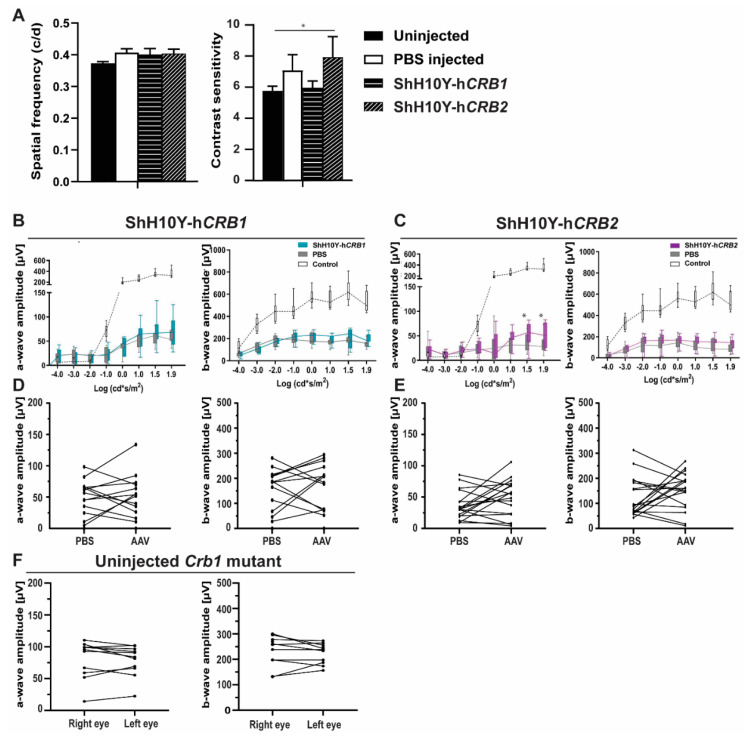
Retinal function in 3M rats after intravitreal injection at P5 of ShH10Y-h*CRB1* or ShH10Y-h*CRB2* is not restored. (**A**) OKT spatial frequency and 0.092 contrast sensitivity of 3M rats injected with PBS, ShH10Y-h*CRB1*, or ShH10Y-h*CRB2*. (**B**,**C**) Quantification of all scotopic a- and b-wave ERG at 3M of the group of rats injected with ShH10Y-h*CRB1* (**B**) or ShH10Y-h*CRB2* (**C**) with uninjected eye and a wild-type as the controls. (**D**,**E**) 1.5 stimulus intensity ERG at 3M comparison of individual rats injected with either ShH10Y-h*CRB1* (**D**) or ShH10Y-h*CRB2* (**E**) compared with PBS-injected eyes. (**F**) 1.5 stimulus intensity ERG at 3M comparison of the left and right eye of non-injected *Crb1* mutant rats. Boxes indicate the 25 and 75% quantile range and whiskers indicate the 5 and 95% quantiles, and the intersection of line and error bar indicates the median of the data (box-and-whisker plot). * *p* < 0.05. Number of animals used: ShH10Y-h*CRB1 n* = 7, 3M OKT and *n* = 15 3M ERG; and for ShH10Y-h*CRB2* injected *n* = 15 3M OKT, and *n* = 18 3M ERG. Mean ± SEM.

Based on the subcellular localization of CRB1 and the tropism of the different AAV capsids, we determined that intravitreal injection of ShH10Y is the most suitable viral vector to infect MGCs and explore gene therapy possibilities in *Crb1* mutant rats (Figure 5C,D). In addition, tropism studies in both control and *Crb1* mutant rats show that larger stretches of the retina are transduced when intravitreally injected at P5 with ShH10Y (Figure S3A,B). For that reason, the *Crb1* mutant rats were injected at P5, with ShH10Y either with h*CRB1* (ShH10Y-h*CRB1*) or h*CRB2* (ShH10Y-h*CRB2*) and the other eye was injected with PBS (mock control). Immunohistochemical analysis of 3-month-old *Crb1* mutant rats injected at P3 with ShH10Y-h*CRB1* show expression of hCRB1 above the OLM (Figure S3C). In addition, rats injected with ShH10Y-h*CRB2* reveal a remaining CRB2 localization at the OLM similar to the uninjected condition (Figure S3D). The visual and retinal function were measured by OKT in 3-month-old, and ERG at 2- and 3-month-old *Crb1* mutant rats. No significant differences were observed in visual function, as measured by the OKT spatial frequency, between untreated, PBS-, ShH10Y-h*CRB1-* or ShH10Y-h*CRB2*-treated eyes at 3 months of age (Figure 7A). In addition, no significant difference in retinal function as measured by ERG was observed between ShH10Y-h*CRB1*-treated and PBS-injected eyes (Figure 7B,C). However, an increase in OKT contrast sensitivity at 0.092 c/d spatial frequency was observed in 3-month-old *Crb1* mutant rats injected with ShH10Y-h*CRB2* compared to PBS-injected rats (Figure 7A). In addition, an increased ERG response at high dark-adapted intensities (1.5 and 1.9) on the a-wave amplitude for rod photoreceptor transmission was observed in *Crb1* mutant rats treated with ShH10Y-h*CRB2* (Figure 7C).

When the retinal function within individual rats was analyzed in 2- and 3-months-old *Crb1* mutant rats, no significant differences were observed between ShH10Y-h*CRB1* and their PBS-injected eyes for both the scotopic a- and b-wave (Figure 7D and Figure S3E). In addition, no differences were observed when treated with ShH10Y-h*CRB2* (Figure 7E and Figure S3F). Interestingly, we observed high variations on the scotopic ERG responses between individual animals measured injected with PBS, ShH10Y-h*CRB1* or ShH10Y-h*CRB2* (a-wave range 10–100 µV; b-wave range 50–300 µV; Figure 7D,E); similar variations were observed when the response of the right and left eye of untreated *Crb1* mutant rats were compared (a-wave range 10–100 µV; b-wave range 100–300 µV; Figure 7F), indicating a variability in the scotopic ERG response between different *Crb1* mutant litters. Because of the absence of an enhanced retinal function after gene therapy delivery, we hypothesize that the injection with our viral vectors at P5 does not allow timely expression of the h*CRB1* or h*CRB2* transgenes before the onset of loss of visual or retinal function in the *Crb1* mutant rats.

## 3. Discussion

In this study, we demonstrate (1) progressive retinal degeneration in *Crb1* mutant rats, causing loss of visual and retinal function; (2) ultrastructural localization of CRB1 at the subapical region of MGCs, and CRB2 at the subapical region of MGCs and photoreceptors in control rats; (3) a decrease in CRB1^INDEL^ at the subapical region of MGCs in *Crb1* mutant rats; (4) tropism by subretinal and intravitreal application of AAV5, AAV9 and ShH10Y in control and *Crb1* mutant rats; and (5) AAV-h*CRB* gene therapy at P5 for MGCs of *Crb1* mutant rats does not result in a functional rescue.

The *Crb1* mutant rats exhibit a significant decreased ERG response at 1 month of age in comparison with age-matched control rats, which is in accordance with previously published results comparing the *Crb1* mutant to BN-Harlan rats [10]. We further expand the characterization of the *Crb1* mutant rat strain by showing a reduced OKT spatial frequency response in 3-month-old *Crb1* mutant rats compared to age-matched control rats. Interestingly, although the control rats show a reduced ERG response from 1 month of age onwards, the OKT spatial frequency decline is minor. This discrepancy has been described before, where similar spatial frequencies only declined months after significant photoreceptor and ERG response loss [20].

Here, we observed the first signs of retinal degeneration from P10 in the *Crb1* mutant rat retina, including photoreceptor nuclei protrusions in the photoreceptor segment layers and OLM breaks at foci throughout the entire retina. These disruptions are ultimately resulting in large regions with a complete disorganized ONL and INL lamination in adult *Crb1* mutant rats. Interestingly, the phenotype at P10 in the *Crb1* mutant rats is comparable to previously described *Crb1* mouse models [3,17], but the retinal phenotype in these rats at 1 month of age is much more severe. In addition, in *Crb1* mouse models, the retinal degeneration is limited to the inferior quadrant [3,17], whereas in the *Crb1* mutant rats it is presented throughout the entire retina. These discrepancies could be because of (1) different genetic backgrounds, including species differences; (2) different types of mutations affecting different CRB isoforms [21,22], thereby expressing a distinct CRB1^INDEL^ protein in the *Crb1* mutant rats; or (3) the total expression levels of CRB2 might be significantly lower in new-born rats compared to new-born mice. Decreased levels of CRB2 or dysregulation of other CRB-interacting proteins could result in less stabilization of the adherens junction complex at the OLM, resulting in a more severe phenotype. In addition, transcriptomic analysis identified several dysregulated pathways in *Crb1* mutant rats, such as TGF-β signaling, matrix metalloproteinases, type II interferon signaling, MAPK cascade, inflammatory pathways, regulation of actin cytoskeleton and many more [10]. More research is required to define which factors play a major role in the retinal degeneration in these *Crb1* mutant rats.

SD-OCT imaging allows to follow the retinal degeneration over time in *Crb1* mutant compared to age-matched control rat retinas. Previous research using SD-OCT with *Crb1* rd8 or *Crb1*^lowMGC^ mouse models show typical ocular lesions, such as pseudo-rosette formation [13,23,24]. Here, we observed a relatively healthy retinal lamination in P17 *Crb1* mutant rat retina with some sporadic disruptions at the OLM. At one month of age, the retinal lamination is disrupted throughout the retina, indicated by hyperreflective regions at the ONL. These hyperreflective disruptions are thought to begin in the OLM because of adhesion abnormalities between the photoreceptor and MGCs. Over time, in 2- and 3-month-old rats, an increased degeneration with larger hyperreflective regions, indicating a potential lack of retinal lamination, is observed. Similar results were obtained using histological sections. Like human *CRB1*-RD patients [24], hyperreflective lesions of various sizes were observed in the *Crb1* mutant rat starting at 1 month of age. The hyperreflective ONL and INL in the *Crb1* mutant rats made automated as well as manual segmentation of the layers challenging, particularly at 2 and 3 months of age. Therefore, we quantified the length of the observed disruptions and compared it to the length of the retinal view, hereby distinguishing between the length of disrupted and normal laminated retinas. Quantification of the data showed a significant increase in OLM breaks and disruptions at the OPL in the *Crb1* mutant compared to age-matched control rats at 1, 2 and 3 months of age. Several researchers have shown that quantification of retinal layer size by SD-OCT, compared with histological sections, contain less variations, presumably due to artefacts from the post-mortem processing [25]. Altogether, these data are showing the importance of the SD-OCT imaging over time in *Crb1* mutant rats.

Immunohistochemical analysis reveal the localization of CRB2 at the subapical region (SAR) in control and *Crb1* mutant rats, while canonical CRB1 is only detected at the SAR of control rats. The CRB1 antibody used detects the carboxyl terminus of the CRB1 protein, and our data show that the *Crb1* mutant rats lack the full length CRB1 at the OLM. In control rats, subcellular localization of CRB1 and CRB2 by immuno-electron microscopy revealed the presence of CRB1 at the SAR adjacent to the adherens junctions in MGCs, and of CRB2 at the SAR in both MGCs and photoreceptors. These data correspond well to the localization previously found in mouse studies [26]. In postmortem human cadaver retinas, however, CRB1 localized at the subapical region in both MGCs and photoreceptor. In these studies, CRB2 localized at the SAR in MGCs, with CRB2 localized at vesicles in the photoreceptor inner segments [12,27]. Interestingly, recent data revealed the subcellular localization of both CRB1 and CRB2 at the SAR of MGCs as well as photoreceptors in human iPSC-derived retinal organoids, second trimester human fetal retina and non-human-primate retinas [8,19]. The cellular localization of CRB1 and CRB2 in Brown Norway rats are therefore only similar to mice.

The *Crb1* gene locus is complex with various *Crb1* splice forms and gene products from alternate promoters, such as *Crb1*-B [17,21,28]. Full-length canonical *Crb1* is encoded on 12 exons with the highly conserved CRB1 carboxyl terminus encoded on exon-12, whereas the *Crb1-B* lacks the conserved carboxyl terminus since encoded on 5′-alternate exon-5a, 6–11 alternate-3′ [21]. The in-frame insertion-deletion mutation in exon 6 of the *Crb1* mutant rat also affects the *Crb1-B* gene, and it is to be tested whether the mutation specifically in *Crb1-B* contributes to the observed early-onset severe retinal phenotype. Interestingly, *Crb1* rd8 mice have an out-of-frame base-pair deletion in exon-9 that causes an alternate-3′ and encodes, therefore, for two truncated transcripts (upstream promoter driving expression of *Crb1* exons 1–6 indel, 7–9 alternate-3′; and a promoter in intron-5 driving expression of *Crb1* exons 5a-6indel, 7–9 alternate-3′) [17]. However, the mutation affecting these two gene products from *Crb1* rd8 do not result in a severe retinal degeneration as observed in the *Crb1* mutant rats, suggesting the existence of other modifying factors that play a role in the severity of the retinal phenotype. Reduced levels of CRB2 in MGCs and/or photoreceptors or retinal progenitors in mice lacking CRB1 result in a significant more severe retinitis pigmentosa or Leber congenital amaurosis retinal phenotype [8,27]. Altogether, these data suggest the existence of other modifying factors that play a role in the severity of the retinal phenotype observed in *Crb1* mutant rats. It remains of interest to test whether low levels of CRB2 contribute to the early-onset severe retinal phenotype observed in new-born *Crb1* mutant rats, whereas high levels of CRB2 might suppress the onset of retinal phenotype in *Crb1* mice.

For AAV-mediated gene therapy purposes, in new-born *Crb1* mutant rats, we determined the retinal tropism of three different AAV serotypes (AAV5, AAV9, ShH10Y) upon different routes of AAV delivery. The AAV capsids were injected at different time points (P5 or P8), either subretinal or intravitreal, in both control and *Crb1* mutant rats. Even though retinal injection efficiency can vary between animals, the transduced layers of cells with a specific capsid and type of injection was similar between animals injected at the two different time points. With subretinal delivery, the serotypes AAV5 and AAV9 successfully transduced the RPE and photoreceptors in new-born control and *Crb1* mutant rats, while subretinal delivery of ShH10Y was transducing RPE, photoreceptors, MGCs and other cell types in the INL. Subretinal delivery of AAV9 at P5 and P8 in both control and *Crb1* mutant rats results in a similar transduction pattern as observed by others after subretinal injection in two-month old Sprague-Dawley rats [29]. In addition, AAV5 tropism at P5 and P8 in control and *Crb1* mutant rats is similar as described in 6- to 8-week-old C57/BL6 wild-type mice [30]. When AAV.CMV.*GFP* vectors were injected intravitreally in new-born control and *Crb1* mutant rats, serotypes AAV5 and AAV9 showed a relatively poor transduction efficiency of both photoreceptors and other cells in the INL. Other researchers showed a poor transduction of cells in the INL as well upon intravitreal delivery of AAV2, AAV6 and AAV8 in two-month-old Sprague-Dawley rats [29]. However, we observed efficient transduction of mainly MGCs in the INL after intravitreal injection in new-born control and *Crb1* mutant rats with ShH10Y, which is consistent with previously published data obtained in the adult rat [31].

Transduction efficiency in the degenerated retina might differ from that in the healthy control retina [30,32]. However, we did not observe a difference in cellular tropism nor in the transduction efficiency between control and *Crb1* mutant rats with all serotypes and the expression vector AAV.CMV.*GFP* tested. Moreover, no differences in tropism were observed when all AAV serotypes were injected either at P5, before the onset of retinal degeneration or at P8, closer to the first signs of degeneration. This might be explained by the correctly laminated retina in the new-born *Crb1* mutant rat retina, and it is well possible that AAV tropism might differ when injected at later time points where the degeneration is more advanced. In summary, we highlight the important differences of AAV capsids and types of delivery that could be considered with future gene therapy approaches.

Finally, we explored the possibility of AAV-mediated h*CRB* gene therapy in *Crb1* mutant new-born rats. Based on the tropism results, we used intravitreal injection of ShH10Y-h*CRB1* and ShH10Y-h*CRB2* at P5 in *Crb1* mutant rats for our gene therapy experiments. The efficacy of these ShH10Y-h*CRB* gene therapy vectors was shown previously [12,13]. When individual untreated *Crb1* mutant rat ERG responses were analyzed at different time points, no differences were detected between the left and right eyes within the same animal. This suggests that both eyes show similar rates of loss of retinal function.

Lack of an improved visual function after AAV-*CRB* application could be caused by the time lag for AAV-mediated gene therapy; it might take several days to weeks before the h*CRB* transgene in *Crb1* mutant rats is expressed at full level in the target cells, in this case the target cells being the MGCs. In our tropism studies, we observed upon intravitreal injection of 1 µL 1 × 10^13^ gc/mL ShH10Y-h*CRB* at P5 a good but potentially incomplete transduction of *Crb1* mutant rat MGCs. In other studies, an increased b-wave was observed when 10-day-old Royal College of Surgeons rats were subretinally injected with a total of 8 µL containing 4 × 10^8^ particles (4 µL superior hemisphere and 4 µL in the inferior hemisphere) of AAV2.CMV.*Mertk* to target defective RPE [33]. Whereas, in our *Crb1* mutant rat studies, 1 µL of a dose of 1 × 10^13^ gc/mL was injected intravitreally at P5, which might be a too low dose of ShH10Y-h*CRB1* or ShH10Y-h*CRB2* to slow down the retinal degeneration observed in the *Crb1* mutant rats. In addition, 1 µL of our gene therapy vector might not spread well throughout the entire retina. Improving the surgical application technique to enlarge the transduced area of the retina could be considered [34]. Moreover, we observed a wide variability in ERG response between different *Crb1* mutant rat litters, likely because of the Brown Norway genetic background. Backcrossing these rats into a more defined genetic background might decrease the observed variability in retinal phenotype. In *Crb1*^rd8/rd8^ mice on a C57BL/6 genetic background, considerable variation in retinal phenotype was observed as well [35]. In addition, the injection efficiency and thereby the number of AAV viral particles taken up by the retinal cells might vary between animals. Moreover, there might be differences in intracellular release of AAV-DNA from capsids inside the targeted cells. Finally, proof-of-concept studies showed functional and structural preservation in a *Crb1* mouse model by using AAV2/9.CMV.h*CRB2* [12] or AA2/ShH10Y.CMV.h*CRB2* [13]. However, the retinal phenotypes in these mice were less severe than the one observed at 1 and 3 months of age *Crb1* mutant rats. In previous studies, we showed that human CRB2 can compensate for the loss of endogenous Crb proteins in the mouse retina [12,13]. Here, we tested human *CRB1* or *CRB2* in gene therapy studies to compensate for the loss of functional Crb1 protein in the rat retina. We showed expression of h*CRB1* in *Crb1* mutant rats after intravitreally injected ShH10Y-h*CRB1* (Figure S3C), but we did not observe an enhanced retinal function after gene therapy delivery. In silico analysis show that both CRB1 and CRB2 are highly conserved proteins between rat and human (Figure S4). Despite the high sequence similarity, proteins may have distinct species-specific properties or activity that need to be taken into account when designing gene therapy studies. Here, we did not test the application of rat *Crb1* or *Crb2* gene therapy vectors to the rat retina due to ethical issues, as the expected outcome of such experiments on a large group of rats is presumed to be negative. Therefore, we cannot exclude the possibility that rat Crb1 or Crb2 proteins could alleviate the loss of endogenous rat Crb1 in models with slower onset of retinal degeneration. All these differences could explain the lack of functional rescue observed here; thus, future experiments could focus on the development of novel gene therapy vectors that allow immediate early-onset expression of transgenes at P5 or low molecular weight drug therapy in new-born rats, or in utero gene therapy approaches.

In conclusion, we further characterized the early-onset morphological phenotype and the retinal function of *Crb1* mutant rats in comparison to age-matched control rats. In addition, we show that the ultrastructural localization of endogenous CRB1 and CRB2 in the rat retina is similar as observed in the mouse retina. In addition, as little is known about AAV tropism administered in new-born Brown Norway rats, we showed differences in cellular tropism when three different AAV capsids were injected in both *Crb1* mutant and control rats. Finally, no timely rescue of the retinal phenotype was observed using retinal function and visual acuity, suggesting the need for earlier onset of expression of recombinant hCRB proteins in Müller glial cells to rescue the severe retinal phenotype in *Crb1* mutant rats.

## 4. Materials and Methods

### 4.1. Animals

Procedures concerning animals were performed in accordance with the EU Directive 2010/63/EU for animal experiments and with permission of the Dutch Central Authority for Scientific Procedures on Animals (CCD), permit number 1160020172924, approved 18, January, 2018. The animals were maintained on a 12 h day–night cycle and were supplied ad libitum with food and water. Brown Norway rats from Janvier Labs with a spontaneous mutation in the *Crb1* gene were used in this study [10]; the *Crb1* mutant rat breeding was set up within the LUMC animal facility. Age-matched control Brown Norway rats lacking the mutation in the *Crb1* gene from Charles River Laboratories were used as controls. Animals were killed by carbon dioxide inhalation.

### 4.2. DNA Isolation and Genetic Analysis

The presence of spontaneous in-frame insertion-deletion (indel) in exon 6 of the *Crb1* gene was validated using DNA extraction from rat’s tails by proteinase K digestion overnight at 55 °C in lysis buffer (100 mM Tris-HCl, pH 8.5, 5 mM EDTA, 0.2% SDS, and 200 mM NaCl). Biopsies were centrifuged for 15 min, the supernatant was transferred and mixed vigorously with isopropanol, followed by a 10 min centrifuge at 14,000 rpm and removal of the supernatant. Then, the pellet was washed twice with 80% ethanol, air dried for 10 min at 55 °C and subsequently resuspended in 200 μL 10% TE (10 mM Tris-HCl, pH 8.0, 1 mM EDTA). Finally, DNase was inactivated for 15 min at 65 °C. For genotyping, PCR with primers targeting the location of the INDEL in the *Crb1* gene was performed and was subsequently sequenced using Sanger Sequencing, FW: 5′-TTCAGACTGTTCAGCCAAATGC-3′, REV: 5′-TGTCCCCATTGGTAAGCCACC-3′.

### 4.3. Electroretinography (ERG)

Dark- and light-adapted ERGs were performed under dim red light using an Espion E2 (Diagnosys, LLC, MA). ERGs were performed on 1, 2 and 3-month-old control and *Crb1* mutant rats. Rats were anesthetized using 100 mg/kg ketamine and 10 mg/kg xylazine intraperitoneally and the pupils were diluted using tropicamide drops (5 mg/mL). Rats were placed on a heating pad, and reference and ground platinum electrodes were placed subcutaneously and in the base of the tail, respectively. ERGs were recorded from both eyes using gold wire electrodes. Hypromellose eye drops (3 mg/mL, Teva) were given between recordings to prevent eyes from drying. Single (scotopic and photopic ERG) or brief train (Flicker ERG) white (6500k) flashes were used. The band-pass filter frequencies were 0.3 and 300 Hz. Scotopic recordings were obtained from dark-adapted animals at the following light intensities: -4, -3, -2, -1, 0, 1, 1.5 and 1.9 log cd s/m2 [36]. Flicker recordings were obtained under a fixed light intensity of 0.5 log cd s/m2 with varying frequency (0.5, 1, 2, 3, 5, 7, 10, 12, 15, 18, 20 and 30 Hz) [37,38]. Photopic recordings were performed following 10 min light adaptation on a background light intensity of 30 cd*m2 and the light intensity series used was: -2, -1, 0, 1, 1.5, 1.9 log cd*s/m2 [36]. The numbers of rats used per time point are indicated under the designated figure. For the gene therapy studies, responses of the treated eye, either right or left eye, were compared with the other eye, mock injected (PBS), at each time point analyzed.

### 4.4. Optokinetic Tracking Reflex (OKT)

Spatial frequency and contrast sensitivity thresholds were measured using an optomotor system (Cerebral Mechanics, Lethbridge, AB, Canada). Two and three month(s)-old rats were placed on a small platform in the center of four computer monitors that formed a virtual drum with a rotating vertical sine wave grating (12°/s (d/s)), as described previously [14]. Head movements in the same direction as the rotating gratings were considered as positive responses and no response was considered as negative response. Spatial frequency thresholds were determined with an increasing staircase paradigm, starting at 0.042 cycles/deg (c/d) with 100% contrast. Contrast sensitivity thresholds were measured across three spatial frequencies (0.092 c/d). The reciprocal of the contrast sensitivity threshold was used as the contrast sensitivity value at each spatial frequency.

### 4.5. Morphological Analysis

Eyes were collected at a range of time points from P5 to 3-month-old control and *Crb1* mutant rats (*n* = 2–4/age/group). For morphological analysis, eyes were enucleated and fixed with 4% paraformaldehyde in phosphate buffered saline (PBS) for 20 min at room temperature. After fixation, the eyes were dehydrated for 30 min in 30, 50, 70, 90 and 99% ethanol. Subsequently, the eyes were embedded in Technovit 7100 (Kulzer, Wehrheim, Germany) and sectioned (3 μm) as previously described [39]. Slides were dried, counterstained with 0.5% toluidine blue and mounted under coverslips using Entellan (Merk, Darmstadt, Germany). Eye sections were scanned using a Pannoramic 250 digital slide scanner (3DHISTECH Ltd., Budapest, Hungary) and images were processed with CaseViewer 2.1 (3DHISTECH Ltd., Budapest, Hungary).

### 4.6. Immunohistochemical Analysis

Eyes were collected at a range of time points from P5 to 3-month-old control and *Crb1* mutant rats (*n* = 2–4/age/group). For immunohistochemical analysis, eyes were enucleated and fixed with 4% paraformaldehyde in PBS for 20 min at room temperature. Then, the eyes were cryo-protected with 15% and 30% sucrose in PBS, embedded in Tissue-Tek O.C.T Compound (Sakura, Finetek), and used for cryosectioning. Cryosections of 8 µm were made with a Leica CM1900 cryostat (Leica Microsystems).

Sections for immunohistochemistry were blocked for 1 h at RT in 10% normal goat serum, 0.4% Triton X-100 and 1% bovine serum albumin (BSA) in PBS. The primary antibodies were diluted in 0.3% normal goat serum, 0.4% Triton X-100 and 1% BSA in PBS and incubated in a moist chamber overnight at 4 °C. After rinsing in PBS, the sections were incubated for 1h at RT with the fluorescent-labelled secondary antibodies goat anti-mouse, goat anti-rabbit or goat anti-chicken IgGs conjugated to Alexa 488, Alexa 555 (1:1000; Abcam) or Cy3 (1:500), which were diluted in 0.1% goat serum in PBS. Nuclei were counterstained with DAPI and mounted in a Vectashield Hardset mounting medium (H1500 or H1800, Vector Laboratories, Burlingame, USA). Sections were imaged on a Leica TCS SP8 confocal microscope. Confocal images were processed with Leica Application Suite X (v3.7.0.20979).

The following primary antibodies were used: P120-catenin (1:250; BD Biosciences Cat# 610134), CRB1 AK2 (1:200; homemade), CRB2 SK11 (1:200; [3]), glutamine synthetase (GS) (1:250; BD Biosciences Cat# 610518), PALS1 (1:200; homemade), recoverin (1:500; Millipore Cat# AB5585) and rhodopsin (1:500; Millipore Cat# MAB5356), SOX9 (1:250; Millipore Cat# AB5535).

### 4.7. Spectral Domain Optical Coherence Tomography (SD-OCT)

P17, 1, 2 and 3 month control and *Crb1* mutant rats were anesthetized using 60 mg/kg ketamine and 60 mg/kg xylazine (50 mg/kg ketamine and 5 mg/kg xylazine for P17 rats) intraperitoneally and the pupils were diluted using tropicamide drops (5 mg/mL). Anesthetized rats were placed in front of the SD-OCT imaging device (EnvisuTM R2210 VHR, Leica, USA). Eyes were kept moisturized with Vidisic Carbogel and Systane ultra-eyedrops during the whole procedure. Image acquisitions were performed using the following parameters: rectangular scans of 3.2 mm by 3.2 mm, A-scans/B-scans: 1000, B-scans: 100, Frames/B-scan: 6 (for high resolution B-scans); and A-scans/B-scans: 400, B-scans: 400, Frames/B-scan: 4 (also known as the isotropic scan for an enface projection image). Thickness of retinal layers were manually measured using Bioptigen InVivoVue Reader and Diver software in the individual layers at 0.3, 0.6, 0.9 and 1.3 mm both sides from the center of the optic nerve head in the nasal–temporal direction. In addition, the length of disrupted and healthy retinal lamination was measured using Fiji ImageJ software, where the frame with the optic nerve head and 800 µm before and after the optic nerve head in nasal-temporal directions were used for quantification. Values of the three different frames in the left and right eye were averaged and plotted in the figure together; so, one value per animal.

### 4.8. Immuno-Electron Microscopy

Immuno-electron microscopy was performed as previously described [40]. In brief, 40 µm sections were incubated with a primary antibody for 48h, it was then incubated with a secondary peroxidase anti-peroxidase for 4 h. After that, sections were developed in a 2,2-diaminobenzidine solution for 4–8 min, and then the gold-substitute-silver-peroxidase method was applied. Sections were then prepared for electron microscopy and overlapping images were collected using a One View Camera (Gatan) as previously described [41].

### 4.9. Delivery of the AAV

For tropism experiments, five days old rats were anesthetized using hypothermia and eight days old rats were anesthetized using an intraperitoneally injected with 35 mg/kg ketamine and 35 mg/kg xylazine. Eyelids were opened and eyes were popped out using surgical tools, the pupils were dilated with 1% tropicamide drops (5 mg/mL) and kept moist with Hypromellose drops. Under visualization with an operating microscope intravitreal and subretinal injections were performed in control and *Crb1* mutant rats. We used AAV2/5, AAV2/9 or AAV2/ShH10^Y445F^, with the full-length CMV promotor, *GFP* and bovine growth hormone polyadenylation sequence for our tropism study. For each serotype and route of delivery, a dose of ~1 × 10^13^ gc/mL was injected in a volume of 1 µL using a 33-gauge blunt-tipped Hamilton syringe (Hamilton Company, Reno, NV, USA). Eyes were closed and protected with “Hansaplast liquid protection” and treated with ointment containing chloramphenicol to prevent infections.

For the gene therapy experiments, similar procedures were followed as described above. Here, one eye was treated with an AAV vector and the other eye was mock-injected (PBS); this was randomized in the left and right eye. We used intravitreal injection of ShH10Y capsids to package the AAV2 inverted terminal repeats, the full length or minimal CMV promoter (CMV, or CMVmin), h*CRB1* or h*CRB2* cDNAs and synthetic poly-adenylation sequence for our gene therapy experiments. The AAVs were spiked with 1/10 dose ShH10Y, with the full-length CMV promotor, *GFP* cDNA and bovine growth hormone polyadenylation sequence. We carefully examined the quality of our AAV-h*CRB* vector preparations by qPCR and Western blots; we recently showed before that these batches of AAV vectors worked efficiently in h*CRB* gene therapy studies [13].

### 4.10. Tropism Quantification

Immunohistochemical slides for tropism studies were imaged on a Leica TCS SP8 confocal microscope, and sections were scanned for AAV-*GFP*-positive areas to re-confirm the subretinal or intravitreal injection and define the tropism. For each condition, 4–8 confocal images spanning the area of transduction of each eye was acquired (*n* ≥ 2 independently injected eyes for each condition). Each individual confocal image was acquired at 40X magnification. The number of eGFP-positive cells within each layer were manually counted. The total number of eGFP-positive cells per eye were divided by transduction diameter to determine the number of positive cells per 100 μm. Then eyes were averaged and coded as - = 0 GFP+ cells; +/−; 1 GFP+ cells, +; 2–5 GFP+ cells, ++; 6–10 GFP+ cells, +++; 11–15 GFP+ cells, ++++; and >16 GFP+ cells.

### 4.11. Statistical Analysis

We performed statistical analysis for group comparisons: comparing the untreated *Crb1* mutant and age-matched control rats by ERG using a two-way ANOVA; comparing all AAV-treated eyes with the other untreated or PBS-injected eyes using a two-way ANOVA (at 2 and 3 months of age); comparing the AAV-treated eye with the other untreated or PBS-injected eyes using a paired t-test; and, finally, statistical analysis on SD-OCT quantifications using a two-way ANOVA. These statistical analyses were performed using GraphPad Prism version 8 (GraphPad Software). All values are expressed as the mean ± SEM if not otherwise indicated. Statistically significant values: * *p* < 0.05; ** *p* < 0.01; *** *p* < 0.001; **** *p* < 0.0001.

## 5. Patents

The LUMC is the holder of patent application PCT/NL2014/050549, which describes the potential clinical use of CRB2; J.W. is listed as an inventor on this patent, and J.W. is an employee of the LUMC.

## Figures and Tables

**Table 1:** Summary of the retinal tropism of three different adeno-associated viral vector (AAV) serotypes, namely, AAV5, AAV9 and ShH10Y, injected via two different routes of delivery at P5 or P8 in control and *Crb1* mutant rat retina. For each serotype and route of delivery, a dose of ~1 × 10^13^ gc/mL was injected in a volume of 1 µL. Quantified data are presented as follows: no GFP positive (-); 1 GFP positive (+/−); 2–5 GFP positive (+); 6–10 GFP positive (++); 11–15 GFP positive (+++); and ≥16 GFP positive cells (++++) per 100 µm. Number of animals analyzed: AAV5 control P5 SR *n* = 6, P5 IV *n* = 2, P8 SR *n* = 2, P8 IV *n* = 2, AAV5 *Crb1* mutant P5 SR *n* = 4, P5 IV *n* = 3, P8 SR *n* = 2, P8 IV *n* = 5; AAV9 control P5 SR *n* = 3, P5 IV *n* = 2, P8 SR *n* = 3, P8 IV *n* = 3, AAV9 *Crb1* mutant P5 SR *n* = 4, P5 IV *n* = 3, P8 SR *n* = 2, P8 IV *n* = 3; ShH10Y control P5 SR *n* = 2, P5 IV *n* = 6, P8 SR *n* = 2, P8 IV *n* = 5, ShH10Y *Crb1* mutant P5 SR *n* = 3, P5 IV *n* = 4, P8 SR *n* = 2, P8 IV *n* = 3. DOI = date of injection; TOI = type of injection; GCL = ganglion cell layer; RNFL = retinal nerve fiber layer; INL = inner nuclear layer; ONL = outer nuclear layer; RPE = retinal pigment epithelium; SR = subretinal injection; IV = intravitreal injection.

AAV	DOI	TOI	GCL/RNFL	INL	ONL	RPE	
AAV5	P5	SR	-	-	++	+	Control
	P5	SR	-	-	+	+	*Crb1* mutant
	P5	IV	-	+/−	+/−	-	Control
	P5	IV	-	-	+	-	*Crb1* mutant
	P8	SR	-	+/−	+++	+	Control
	P8	SR	-	-	+++	+	*Crb1* mutant
	P8	IV	-	-	+	-	Control
	P8	IV	-	+	+/−	-	*Crb1* mutant
AAV9	P5	SR	-	-	++	+	Control
	P5	SR	-	-	+++	+	*Crb1* mutant
	P5	IV	-	++	+/−	-	Control
	P5	IV	+/−	+	-	-	*Crb1* mutant
	P8	SR	-	-	+++	+	Control
	P8	SR	-	-	+++	+	*Crb1* mutant
	P8	IV	-	+/−	-	-	Control
	P8	IV	-	+/−	-	-	*Crb1* mutant
ShH10Y	P5	SR	-	+	+	+	Control
	P5	SR	-	+	+	+	*Crb1* mutant
	P5	IV	-	+++	+/−	-	Control
	P5	IV	-	++	+	-	*Crb1* mutant
	P8	SR	-	+	+	+	Control
	P8	SR	-	+	+	+	*Crb1* mutant
	P8	IV	-	++	-	-	Control
	P8	IV	-	++	+/−	-	*Crb1* mutant

## Data Availability

The data that support the findings of this study are available from the corresponding author upon reasonable request.

## Supplementary Materials

The following are available online at https://www.mdpi.com/article/10.3390/ijms22073563/s1. Figure S1. Retinal function further decreases in 3 and 5 months of age *Crb1* mutant compared to control rats. Quantitative analysis of the scotopic a-wave (A) and b-wave (B), the photopic b-wave (C), the b-wave/a-wave amplitude and b/a-ratios (b/a) (D), and the flicker amplitude response (E) in 3 and 5 months of age *Crb1* mutant and control rats. Boxes indicate the 25 and 75% quantile range and whiskers indicate the 5 and 95% quantiles, and the intersection of line and error bar indicates the median of the data (box-and-whisker plot). Number of animals used for 3 months: control = 8, *Crb1* mutant = 6, and 5M: control = 6, *Crb1* mutant = 6. Mean ± SEM. * *p* < 0.05; ** *p* < 0.01; *** *p* < 0.001; **** *p* < 0.0001. Figure S2. Predominantly MGC transduction upon subretinal or intravitreal delivery of ShH10Y in both control and *Crb1* mutant rat retina. ShH10Y transduction at P5 subretinal (A,B), or intravitreal (C,D), and P8 subretinal (E,F), or intravitreal (G,H) in one month control (A,C,E,G) or *Crb1* mutant (B,D,F,H) rats retina. Co-stained with glutamine synthetase (GS; red), co-localization of GFP and GS indicated with arrowheads. At least *n* = 2 eyes used per time point. INL = inner nuclear layer, ONL = outer nuclear layer. SR = subretinal injection, IV = intravitreal injection. Scale bar: 20 µm. Figure S3. Gene therapy of ShH10Y-h*CRB1* or ShH10Y-h*CRB2* at P5 does not improve the severe retinal phenotype measured by ERG analysis at 2M of age. Overview of control (A) and *Crb1* mutant (B) rats intravitreally injected at P5 with ShH10Y-*GFP*, indicating the area of transduction. Immunohistochemical analysis of 3 months old *Crb1* mutant rats intravitreally injected at P3 with ShH10Y-h*CRB1* (C) or with ShH10Y-h*CRB2* (D) revealing the expression of hCRB1 and hCRB2. And 1.5 stimulus intensity ERG comparison of 2M individual rats injected with either ShH10Y-h*CRB1* (E) or ShH10Y-h*CRB2* (F) at P5 compared with PBS injected eyes. Number of animals used: P5 injection with ShH10Y-h*CRB1 n* = 11; ShH10Y-h*CRB2 n* = 9. INL = inner nuclear layer, OLM = outer limiting membrane, ONL = outer nuclear layer. Scale bar: (A,B) 200 µm, (C,D) 20 µm. Figure S4. Highly conserved CRB1 and CRB2 protein sequence between human and Brown Norway rats. Protein sequence alignment of human CRB1 with rat Crb1 (A) and human CRB2 with rat Crb2 (B). Protein sequence alignment reveals 75% identical matches and 84% conservative substitutions for human CRB1 compared with rat Crb1. And for human CRB2 compared with rat Crb2 there are 77% identical matches and 82% conservative substitutions shown. Upper and bold sequence is human, lower sequence is rat. Sequence alignment data from Uniprot and BLAST.
