# Supplementary material for: Defining Phenotype, Tropism, and Retinal Gene Therapy Using Adeno-Associated Viral Vectors (AAVs) in New-Born Brown Norway Rats with a Spontaneous Mutation in Crb1
Source: Int J Mol Sci. 2021 Mar 30;22(7):3563. doi: 10.3390/ijms22073563 (PMC8036486; doi:10.3390/ijms22073563)
Supplement: Supplementary file 1 [file ijms-22-03563-s001.zip › Figure S legends.docx]

**Figure S1.** **Retinal function further decreases in 3 and 5 months of age *Crb1* mutant compared to control rats.** Quantitative analysis of the scotopic a-wave (A) and b-wave (B), the photopic b-wave (C), the b-wave/a-wave amplitude and b/a-ratios (b/a) (D), and the flicker amplitude response (E) in 3 and 5 months of age *Crb1* mutant and control rats. Boxes indicate the 25 and 75% quantile range and whiskers indicate the 5 and 95% quantiles, and the intersection of line and error bar indicates the median of the data (box-and-whisker plot). Number of animals used for 3 months: control=8, *Crb1* mutant =6, and 5M: control=6, *Crb1* mutant =6. Mean ± SEM. * P < 0.05; ** P < 0.01; *** P < 0.001; **** P < 0.0001.

**Figure S2.** **Predominantly MGC transduction upon subretinal or intravitreal delivery of ShH10Y in both control and *Crb1* mutant rat retina.** ShH10Y transduction at P5 subretinal (A-B), or intravitreal (C-D), and P8 subretinal (E-F), or intravitreal (G-H) in one month control (A, C, E, G) or *Crb1* mutant (B, D, F, H) rats retina. Co-stained with glutamine synthetase (GS; red), co-localization of GFP and GS indicated with arrowheads. At least n=2 eyes used per time point. INL = inner nuclear layer, ONL = outer nuclear layer. SR = subretinal injection, IV = intravitreal injection. Scale bar: 20 µm.

**Figure S3.** **Gene therapy of ShH10Y-h*CRB1* or ShH10Y-h*CRB2* at P5 does not improve the severe retinal phenotype measured by ERG analysis at 2M of age.** Overview of control (A) and *Crb1* mutant (B) rats intravitreally injected at P5 with ShH10Y-*GFP*, indicating the area of transduction. Immunohistochemical analysis of 3 months old *Crb1* mutant rats intravitreally injected at P3 with ShH10Y-h*CRB1* (C) or with ShH10Y-h*CRB2* (D) revealing the expression of hCRB1 and hCRB2. And 1.5 stimulus intensity ERG comparison of 2M individual rats injected with either ShH10Y-h*CRB1* (E) or ShH10Y-h*CRB2* (F) at P5 compared with PBS injected eyes. Number of animals used: P5 injection with ShH10Y-h*CRB1* n=11; ShH10Y-h*CRB2* n=9. INL = inner nuclear layer, OLM = outer limiting membrane, ONL = outer nuclear layer. Scale bar: (A, B) 200 µm, (C, D) 20 µm.

**Figure S4.** **Highly conserved CRB1 and CRB2 protein sequence between human and Brown Norway rats.** Protein sequence alignment of human CRB1 with rat Crb1 (A) and human CRB2 with rat Crb2 (B). Protein sequence alignment reveals 75% identical matches and 84% conservative substitutions for human CRB1 compared with rat Crb1. And for human CRB2 compared with rat Crb2 there are 77% identical matches and 82% conservative substitutions shown. Upper and bold sequence is human, lower sequence is rat. Sequence alignment data from Uniprot and BLAST.
