# Supplementary figures and images for: Defining Phenotype, Tropism, and Retinal Gene Therapy Using Adeno-Associated Viral Vectors (AAVs) in New-Born Brown Norway Rats with a Spontaneous Mutation in Crb1
Source: Int J Mol Sci. 2021 Mar 30;22(7):3563. doi: 10.3390/ijms22073563 (PMC8036486; doi:10.3390/ijms22073563)

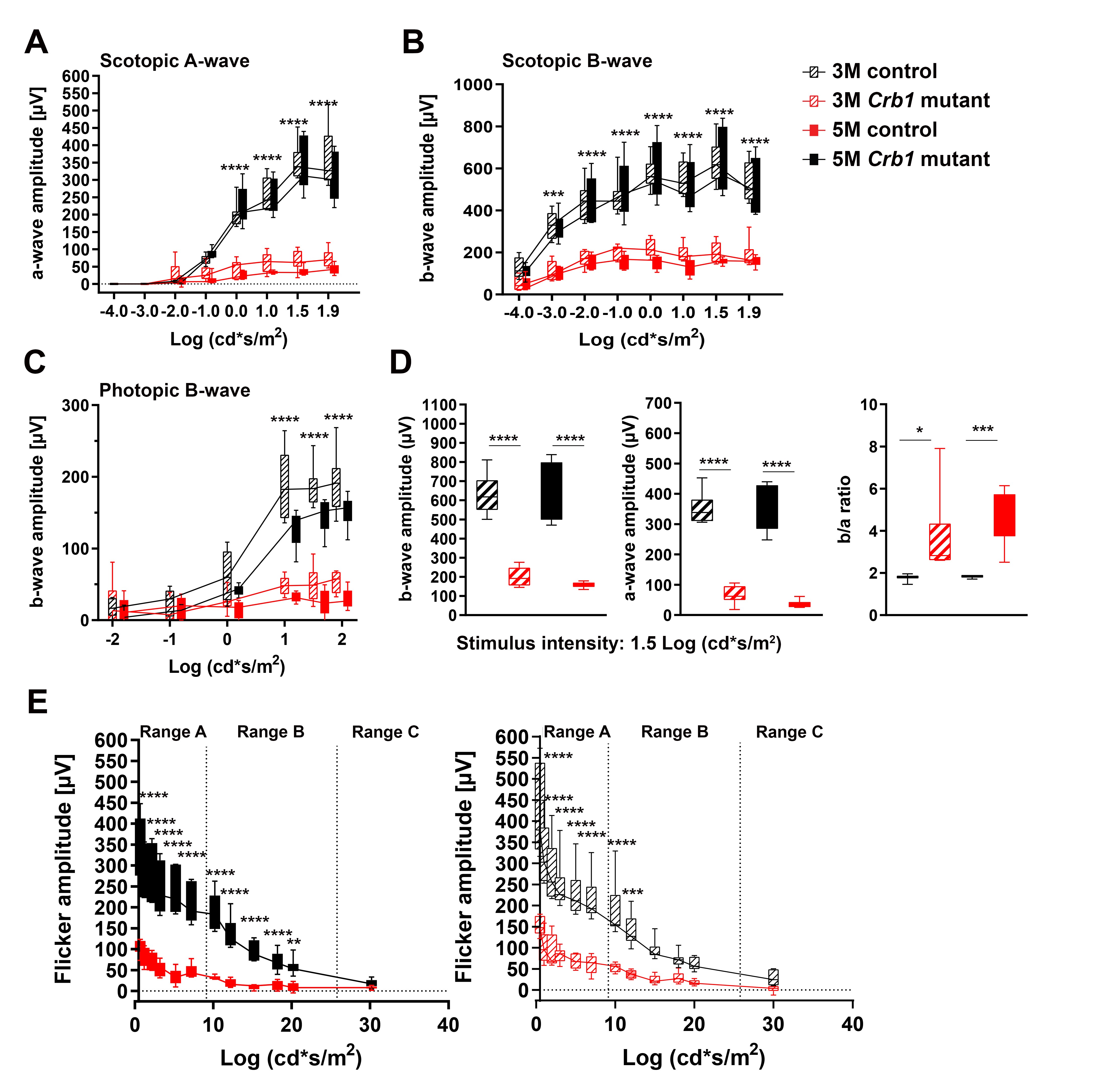

Supplement: Supplementary file 1 [file ijms-22-03563-s001.zip › Figure S1.jpg]

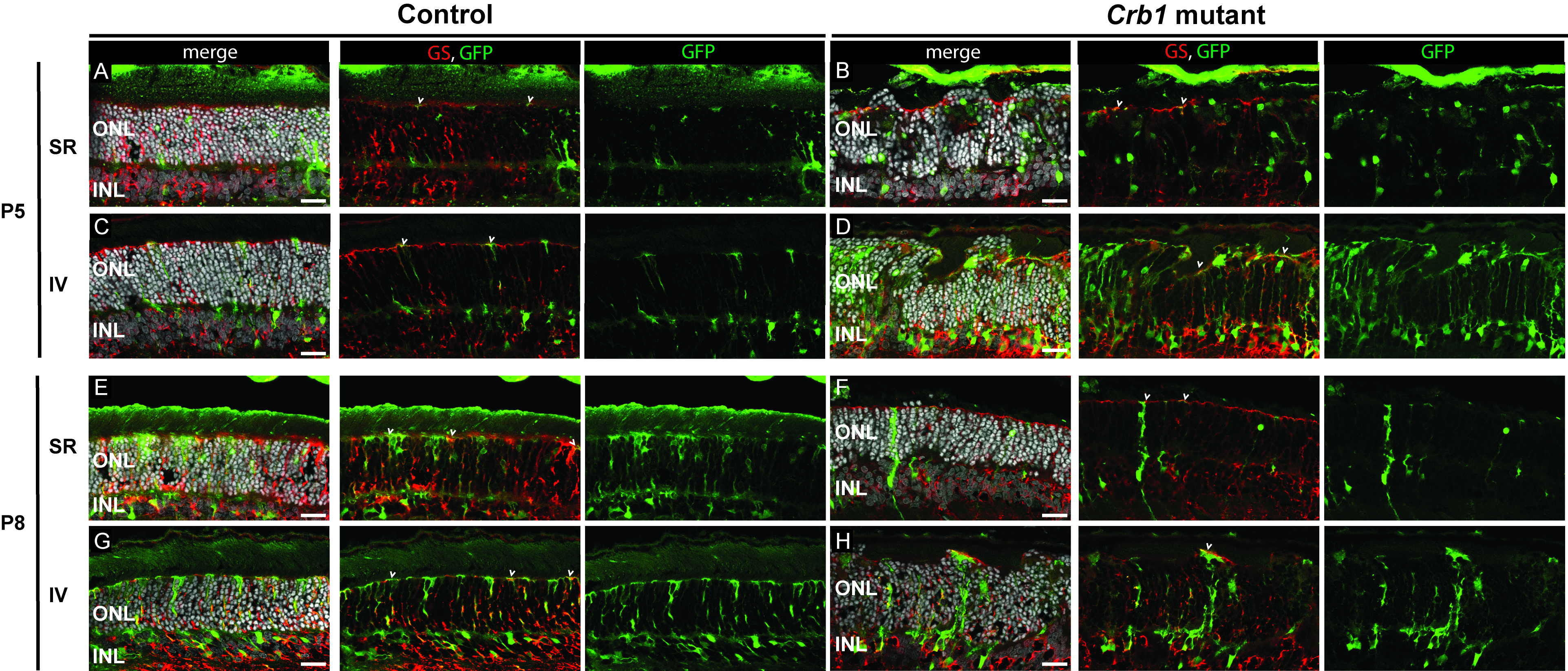

Supplement: Supplementary file 1 [file ijms-22-03563-s001.zip › Figure S2.tif]

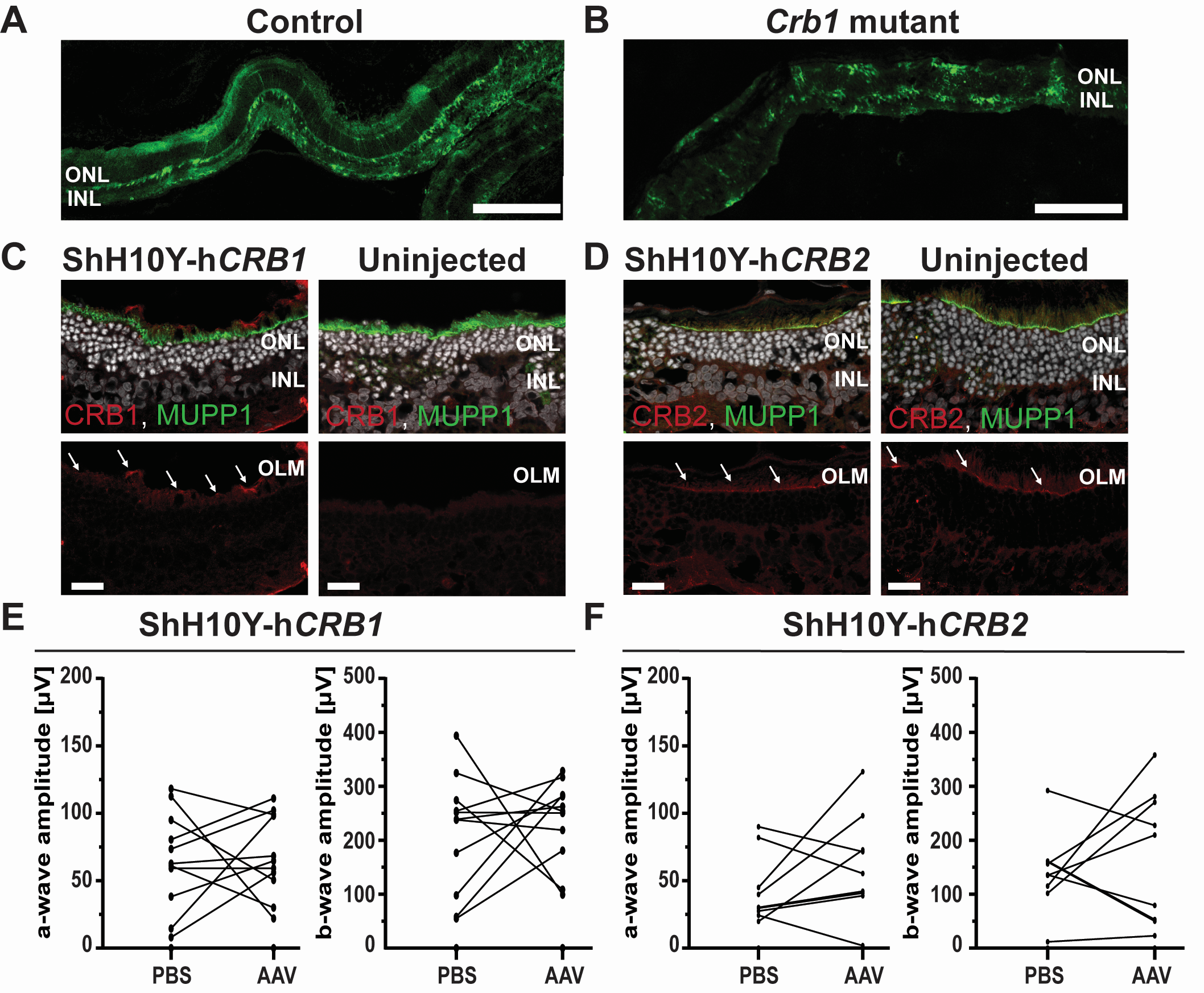

Supplement: Supplementary file 1 [file ijms-22-03563-s001.zip › Figure S3.tif]
